# Quantitative Structure–Activity Relationship Modeling of Kinase Selectivity Profiles

**DOI:** 10.3390/molecules22091576

**Published:** 2017-09-19

**Authors:** Sandeepkumar Kothiwale, Corina Borza, Ambra Pozzi, Jens Meiler

**Affiliations:** 1 Department of Chemistry, Center for Structural Biology, Institute of Chemical Biology Vanderbilt University, Nashville, TN 37232, USA; kothiwale.sandeep@gmail.com; 2 Department of Medicine, Division of Nephrology, Vanderbilt University, Nashville, TN 37232, USA; corina.borza@vanderbilt.edu (C.B.); ambra.pozzi@vanderbilt.edu (A.P.); 3 Department of Medicine, Veterans Affairs Hospital, Nashville, TN 37232, USA

**Keywords:** kinase selectivity profile, quantitative structure–activity relation, BCL::Cheminfo, artificial neural networks

## Abstract

The discovery of selective inhibitors of biological target proteins is the primary goal of many drug discovery campaigns. However, this goal has proven elusive, especially for inhibitors targeting the well-conserved orthosteric adenosine triphosphate (ATP) binding pocket of kinase enzymes. The human kinome is large and it is rather difficult to profile early lead compounds against around 500 targets to gain an upfront knowledge on selectivity. Further, selectivity can change drastically during derivatization of an initial lead compound. Here, we have introduced a computational model to support the profiling of compounds early in the drug discovery pipeline. On the basis of the extensive profiled activity of 70 kinase inhibitors against 379 kinases, including 81 tyrosine kinases, we developed a quantitative structure–activity relation (QSAR) model using artificial neural networks, to predict the activity of these kinase inhibitors against the panel of 379 kinases. The model’s performance in predicting activity ranges from 0.6 to 0.8 depending on the kinase, from the area under the curve (AUC) of the receiver operating characteristics (ROC). The profiler is available online at http://www.meilerlab.org/index.php/servers/show?s_id=23.

## 1. Introduction

**Figure 1:**
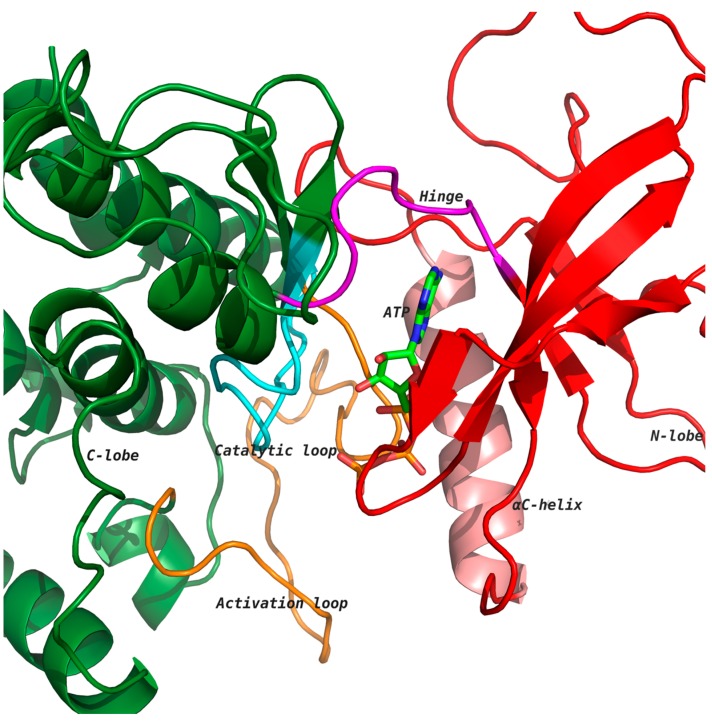
General structure of a kinase domain consisting of two lobes, a helix-rich C-terminal lobe (green) and a sheet-rich N-terminal lobe (red). The ATP binding pocket is present at the interface of the two lobes. The heterocyclic ring of ATP forms hydrogen bonds with the hinge loop (magenta). The activation loop (orange) undergoes a conformation change upon phosphorylation of a conserved residue allowing the substrate to bind. Arginine of the His-Arg-Asp (HRD) motif in the catalytic loop interacts with phosphate in the activation segment. Aspartate of the HRD motif accepts a proton from the substrate hydroxyl group during phosphotransfer mechanism.

Kinases are a large and diverse multigene family involved in the regulation of several cell functions [1,2,3]. Kinases catalyze the transfer of the γ-phosphate group of ATP to selective amino acids, namely, serine, threonine and tyrosine residues, and the selective phosphorylation of target proteins results in either the activation or downregulation of the protein function. Kinases have two distinct lobes: an amino-terminal lobe comprising a five-stranded β-sheet and one α-helix, and a carboxy-terminal lobe that is mainly α-helical (Figure 1). The ATP binding cleft is located at the interface of the two lobes, which is lined with several highly conserved residues [2,4,5]. The heterocyclic ring of ATP interacts with the hinge region through hydrogen bonds (Figure 1). Kinases undergo conformational changes due to ATP binding, leading to the phosphorylation of substrate proteins [4,6]. Most notably, the catalytic and activation loop attain conformations that align important residues involved in the transfer of phosphate from the ATP molecule to a target substrate protein [2,6,7]. Figure 1 shows ATP bound to the kinase domain of the receptor tyrosine kinase (RTK) discoidin domain receptor 1 (DDR1; PDB: 3ZOS) [8] in the active state, which allows for the transfer of the phosphate group from ATP to the phosphorylation site of the activation loop (orange) [4,5]. The aspartate of conserved motif His-Arg-Asp (HRD) in the catalytic loop (cyan) accepts a proton from the substrate hydroxyl group during the phosphotransfer mechanism [5,6].

There are more than 500 kinases in the human genome, and 92 of them belong to the tyrosine kinase family, while the rest belong to the serine/threonine kinase family [9]. These kinases interact with phosphorylate diverse substrates, including other kinases, enzymes, transcription factors, receptors and other regulatory proteins.

Among the tyrosine kinases that have been identified, most are transmembrane receptors, while a small group is comprised of a cytoplasmic non-receptor type (non-RTK) [10]. RTKs are single membrane-spanning receptors that play critical roles in transducing extracellular signals to the intracellular environment [2,10]. RTKs have an extracellular ligand-binding domain, a single transmembrane hydrophobic helix and a cytoplasmic domain containing the kinase domain [10]. The non-RTKs have a kinase domain and often possess several protein–protein interaction domains, such as SH2, SH3, and PH domains [3]. In order to be activated, RTKs need to bind to a ligand that can be a soluble factor or an extracellular matrix protein, such as in the case of DDRs that are activated by collagen. Upon ligand binding, RTKs undergo auto-phosphorylation on tyrosine residues within and outside the catalytic domain, as well as dimerization and/or oligomerization. Upon activation, RTKs can phosphorylate several substrates controlling various cellular processes, including cell proliferation, differentiation, migration, metabolism, programmed cell death, and extracellular matrix homeostasis [11,12]. The activation of non-RTKs involves heterologous protein–protein interactions for enabling trans-phosphorylation [3]. Non-RTKs also couple to receptors that lack intrinsic enzymatic activity and thus rely on these non-RTKs to originate intracellular signaling [6,11].

While the phosphorylation of RTKs and/or non-RTKs is a key step in activating the receptor, their dephosphorylation by selective protein tyrosine phosphatases or their internalization and degradation is needed in order to turn off receptor-mediated signaling. Abnormal RTK phosphorylation due to either increased ligand-dependent and/or -independent receptor phosphorylation or decreased phosphatase-mediated receptor dephosphorylation is often observed in diseases such as cancer, diabetes and fibrosis. Thus, targeting RTKs has been viewed as a promising strategy to dampen receptor-mediated function in disease.

RTKs are pharmacologically targeted by: (a) direct targeting of the kinase catalytic activity by interfering with auto- and trans-phosphorylation, (b) inhibiting activation of the kinases by blocking their dimerization/oligomerization, and/or (c) blocking ligand/receptor interaction in order to prevent receptor activation [13,14]. Small-molecule ATP-competitive inhibitors were the first promising therapeutic strategies targeting the catalytic activity of kinases and have been the target of choice in the small-molecule space. Most small-molecule kinase inhibitors are ATP mimics [9,15,16], as they present one to three hydrogen bonds to residues that normally interact with the adenine ring of ATP. The adenine ring forms two key hydrogen bonds at the N-1 and N-6 positions with the kinase hinge region—the segment connecting the N-terminal and the C-terminal lobe (Figure 1) [17,18]. The ribose binds in the ribose-binding pocket and the triphosphate groups lie in a channel extending to the substrate binding site. Kinases have a conserved activation loop assuming a large number of conformations that regulate access to the ATP binding site, which allows the enzyme to switch between the active and inactive state [4,5]. In the active state, the loop is often phosphorylated, while in the inactive state, it blocks the substrate binding site.

**Figure 2:**
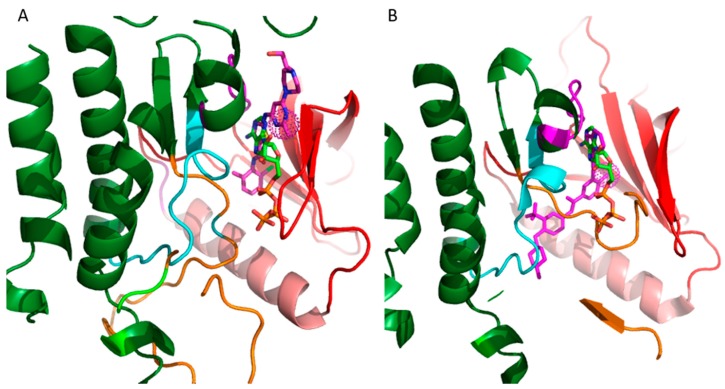
Binding of type I and type II inhibitors in the ATP pocket or receptor tyrosine kinases (RTKs) alongside ATP for comparison. The inhibitors mimic the interactions that the heterocyclic ring of ATP (green) has with the hinge loop (magenta). (**A**) Dasatanib (magenta), a type I inhibitor, is shown in the binding pocket of Abl2 kinase domain (PDB: 4XLI) locked in active state. The activation loop (orange) is positioned such that it is phosphorylated and is able to recruit substrate proteins. The catalytic loop (cyan) enables the transfer of the phosphate group to the substrate protein; (**B**) Pontatinib, a type II inhibitor, is shown bound to the kinase domain of DDR1 kinase (PDB: 3ZOS) locked in an inactive state. ATP (green) is bound in the active state and is shown here for comparison to Figure 2A. The conformation of the activation loop and the catalytic loop differ from those found in the active state.

As of 2015, 28 small-molecule kinase inhibitors have been approved for clinical use, and more are being investigated in clinical trials [16,19,20]. The vast majority of these inhibitors target the ATP binding site. The binding mode of the inhibitors is categorized on the basis of the conformation of a conserved Asp-Phe-Gly (DFG) motif within the activation loop. The type I inhibitors block the kinase in the active conformation of the kinase, as the DFG motif of the activation loop faces into the ATP binding site (Figure 2A). The heterocyclic ring of such inhibitors occupies the adenine binding site while the other parts of the molecule occupy the adjacent hydrophobic regions I and II. Examples include the US Food and Drug Administration (FDA)-approved dasatinib used to target Abelson related kinase (Abl2) in chronic myeloid leukemia (CML). Type I inhibitors have high cross-reactivity within the kinase family because of a high degree of sequence and structural similarity in the ATP binding site. In general, type I inhibitors tend to be promiscuous, as they target the well-conserved ATP binding sites in the active conformation of the kinase. Figure 2A shows superimposed binding poses of dasatinib (magenta sticks) and ATP (green sticks) into the Abl2 kinase (PDB: 4XLI) domain. The heterocyclic ring of dasatinib occupies the ATP purine binding site that serves as a scaffold for side-chains occupying the hydrophobic site I near the pocket shown by the spherical magenta dots.

Type II inhibitors bind the inactive conformation of the kinase, in which the DFG motif faces outward such that the aspartate side chain faces out to the solvent. The 180° rotation opens up an additional hydrophobic pocket, the “specificity pocket”, which is exploited by type II inhibitors. Type II inhibitors tend to be more selective, because the inactive “DFG-out” kinase conformation allows additional interactions between the inhibitor and specific, not-well-conserved exposed hydrophobic sites within the kinase domain. Examples include the FDA-approved imanitib and ponatinib against Abl2 and Bcr-Abl in CML. Figure 2B shows ponatinib (magenta) bound to the inactive state of DDR1 kinase (PDB: 3ZOS). The allosteric site that the type II inhibitors target is shown by the spherical magenta dots.

As the kinase inhibitors target the orthosteric and well-conserved ATP binding pocket, they are multi-targeted and often inhibit a large number of kinases in a non-specific manner [15]. Improved tyrosine kinase selectivity is a major challenge for developing promising lead compounds into therapeutics because of the side-effects caused by off-target activity. Dasatinib is a potent type I kinase inhibitor and is effective in patients with imatinib-resistant CML. However, in addition to Abl, it inhibits several other kinases, including C-Kit, platelet derived growth factor (PDGF) receptor, and ephrin receptors. Another example is sunitinib, a dual vascular endothelial growth factor (VEGF) and PDGF receptor inhibitor approved by the FDA for the treatment of renal cell carcinoma. This inhibitor, however, also inhibits C-kit and AMPK, thus accounting for some of its cardiovascular toxicity. The degree of cross-reactivity has been determined by a number of studies, which report inhibitor activities against a large panel of kinases, including the studies of Davis and colleagues, who screened a total of 70 known inhibitors against a panel of 379 kinases in a competition binding assay [15].

In order to devise more selective kinase inhibitors, it is desirable to profile highly potent inhibitors for kinase selectivity early in the hit-to-lead and lead-to-drug optimization process. We hypothesize that computational models could be used to predict a hit compound’s kinase activity profile early in the lead optimization process. Further, in a second step, the selectivity profile for to-be-synthesized derivatives of hit compounds can also be predicted, thereby contributing to the prioritization of hit compounds for hit-to-lead optimization. Several computational approaches have been developed for predicting kinase activity profiles. Sheinerman and colleagues developed a computational approach to design a binding site signature that uses three-dimensional (3D) X-ray structure information of a kinase-inhibitor complex to predict the small-molecule’s selectivity profile [21]. Subramanian and colleagues applied this approach to predict the off-target kinase selectivity profile for 15 molecules against 280 members of the human kinome [22]. A co-crystal structure of the ligand of interest is a pre-requisite for this method. The input data include interacting residues in the binding pocket of the target kinase enzyme. Sciabola and colleagues used the Free-Wilson approach to build quantitative structure–activity relationship (QSAR) models for a series of chemical analogs [23]. The Free-Wilson concept states that the biological activity of a molecule can be described as the sum of activity contributions from specific substructures [24]. A limitation therefore is that it cannot make predictions about functional groups that are not present in the original set of compounds. Subramanium and colleagues reported an average accuracy/sensitivity/specificity of 0.81/0.37/0.93 for 15 kinase inhibitors at an activity cutoff of *K_d_* = 3 µM against a subset of 280 kinases. Sciabola and colleagues also used an in-house scaffold library for their study, reporting a correlation of greater than 0.85 between experimental and predicted IC_50_ values for two series of compounds.

For the present study, we developed QSAR models for predicting the activity profiles of kinase inhibitors against a panel of kinases using an artificial neural network (ANN)-based methodology. The objective of QSAR modeling is to correlate the chemical structure with biological activity in a quantitative way. There are three prerequisites for QSAR modeling: (a) a quantitative description of the molecular structure (descriptor), (b) biological activities of a diverse set of molecules, and (c) a mathematical technique for correlating descriptors to predict activity. Machine learning techniques are commonly applied to develop non-linear mathematical QSAR models. Here, we used ANNs as implemented in BCL::Cheminfo to generate the kinase selectivity models [25].

## 2. Results

ANN QSAR models for predicting kinase selectivity profiles were built using the cheminformatics framework implemented in BCL::Cheminfo. The inhibition data of 70 kinase inhibitors against 379 kinases reported by Davis and colleagues [15] was used to train the ANNs. The chemical structure of each inhibitor was encoded using molecular descriptors. The numeric description was used as the input to the ANNs, and binary experimental kinase activity was used as the output for training. We will first describe the dataset used for building the models, followed by the molecular descriptors used for numerical encoding.

### 2.1. Training Dataset

The ANN QSAR models were trained using kinase inhibitor data published by Davis and colleagues [15]. Davis and colleagues reported the interaction profile of a diverse set of 70 known kinase inhibitors against 379 kinases. The molecules that were tested represented mature inhibitors optimized against specific kinases of interest. The study was performed using ATP site-dependent competition binding assays. Five models were developed using different *K_d_* cutoff values for specifying active molecules: 0.1, 0.5, 1, 3 and 10 µM.

### 2.2. Molecular Descriptors

Chemical structures were encoded using a set of molecular descriptors using BCL::Cheminfo [25,26]. The descriptors were translationally and rotationally invariant geometric functions that described the distribution of molecular properties in the structure (e.g., mass, volume, surface area, partial charge, electronegativity, polarizability, etc.). The descriptors could be grouped into five categories on the basis of the level of information they provided—one dimensional (1D) descriptors were computed as scalar values derived from a molecular formula, for example, molecular weight and total charge. Two-dimensional (2D) descriptors were calculated using molecular connectivity information and included properties such as hydrogen-bond acceptors/donors, the number of ring systems, and approximations of the surface area and volume. Information about the molecular configuration (i.e., connectivity and stereochemistry) was used to calculate 2.5D descriptors. Conformation-dependent or 3D descriptors encode atomic properties (e.g., partial charge and polarizability) in a 3D fingerprint using radial distribution functions (RDF) and 3D autocorrelations (3DA). The molecular descriptors used in this study are described in our earlier publications [25,26].

### 2.3. Artificial Neural Network Model Development and Validation

ANNs in this study contained 400 inputs (a result of encoding the chemical structure with molecular descriptors), 32 hidden neurons, and 1 output neuron for each kinase included in the model. The ANNs were trained using simple back-propagation and a sigmoid transfer function with weight update parameters of η = 0.1 and α = 0.5 [25,26].

### 2.4. Metrics to Evaluate Artificial Neural Network Prediction Accuracy

Five models were generated by using different *K_d_* cutoff values for specifying the active molecules. Each model predicted the activity of a small molecule in terms of 379 binary outcomes for each of the kinase molecules. The binary predictions fell into the following four categories:

True Positives (TP)—Experimentally active, predicted to be active.
True Negatives (TN)—Experimentally inactive, predicted to be inactive.
False Positives (FP)—Experimentally inactive, predicted to be active.
False Negatives (FN)—Experimentally active, predicted to be inactive.

Table 1 shows the overall accuracy (ACC), Matthew’s correlation coefficient (MCC), the sensitivity (SEN) and the specificity/selectivity (SEL) of each model calculated by pooling all TP, FP, TN and FN across all kinases and small molecules. The measures to assess the quality of the predictive models were defined as follows:

Sensitivity (SEN) = TP/(TP + FN)
Selectivity (SEL) = TN/(TN + FP)
Accuracy (ACC) = (TP + TN)/(TP + TN + FP + FN)
Matthews correlation coefficient (MCC) =
  (1) ((TP×TN−FP×FN))⁄√((TP+FP)×(TP+FN)×(TN+FP)×(TN+FN))
Positive predictive value (PPV) = TP/(TP + FP)
Negative predictive value (NPV) = TN/(TN + FN)

## 3. Discussion

**Figure 3:**
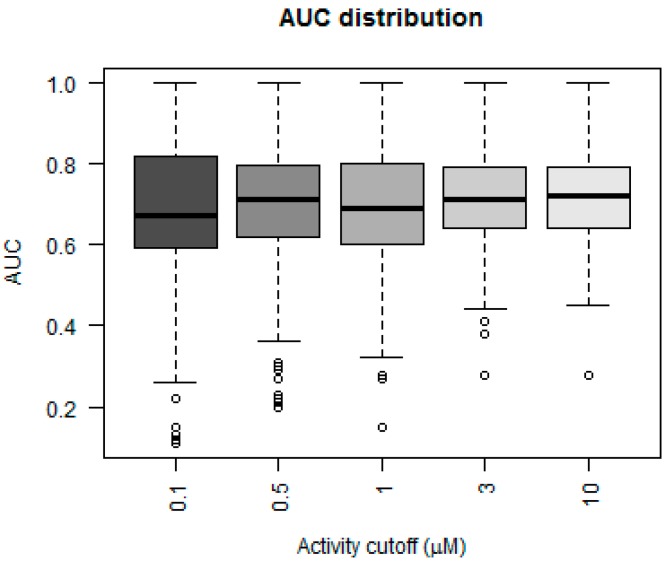
Area under curve distribution. Box plot showing the performance of the five models using distribution of area under the curve (AUC) values for individual kinases. The five models have been generated using activity values specified at different cutoffs as indicated on the x-axis. The upper and lower edges of the box correspond to the first and third quartiles. The horizontal dash in the box represents the median value. The whiskers extend from the edge to the highest/lowest value within the 1.5× interquartile (IQR) of the box, where the IQR is the distance between the first and third quartile.

The three metrics, accuracy, sensitivity and specificity, were very stable for the models using activity cutoff values of greater than 0.5 µM. However, Matthew’s correlation coefficient was highest for a cutoff value of 10 µM, even with a higher number of indicated active kinase-inhibitor pair increases. The prediction accuracy of the models could also be evaluated using values derived from receiver operator characteristic (ROC) curves. A ROC curve plots the true positive rate (TPR; i.e., active molecules predicted as active) versus the false positive rate (FPR; i.e., inactive molecules predictive as active) as a fraction of the total number of known inactive molecules. A TPR versus FPR slope of 1, which results in an area under the curve (AUC) of 0.5, indicates a model that is no better than random at correctly predicting a compound as active versus inactive. An increase in the slope and therefore the area under the curve indicates an increase in the predictive power over a random guess. Figure 3 depicts a box plot showing the performance of the five models in terms of AUC values for each of the 379 kinases.

The AUC value for greater than 50% of the kinases was above 0.75 for the models that considered 3 and 10 µM as their activity cutoff. The models were compared statistically using a Mann–Whitney paired test, to see which model performed better in terms of higher AUC values. The model built using an activity cutoff value of 10 µM performed better than all the other models at a confidence interval of 95%. A Fisher’s test showed that the 10 µM model was statistically significantly better than a random model, which predicted 50% of cases as positive.

**Figure 4:**
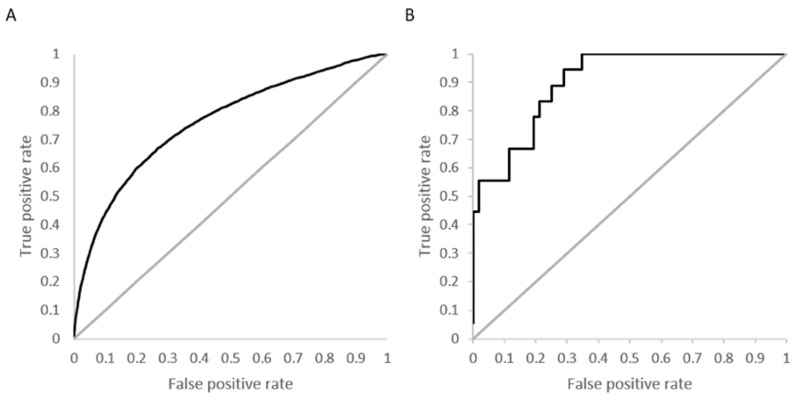
Performance of neural networks for kinase selectivity: (**A**) Overall receiver operator curve (ROC) computed for model developed at activity cutoff of *K_d_* = 10 µM. (**B**) Example of ROC curve for calmodulin-dependent protein kinase-1, which has 18 active molecules at *K_d_* < 10 µM with an area under the curve (AUC) of 0.91.

Figure 4A shows the overall ROC curve for the model developed using activity specified at *K_d_* < 10 µM with a computed AUC of 0.76. Figure 4B is an example ROC curve for a kinase with 18 active molecules and a high prediction accuracy (88%) and specificity (98%). The calculated AUC for this kinase, calmodulin-dependent protein kinase-1, is 0.91.

**Figure 5:**
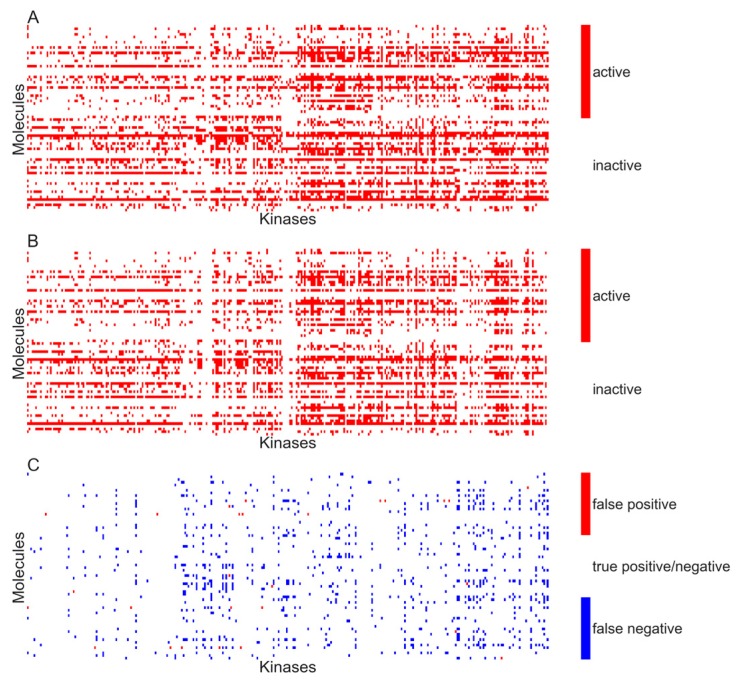
Activity matrix of all inhibitors versus all kinases. (**A**) Experimental activity matrix as reported by Davis and colleagues. The pixels in red correspond to active molecules at 10 µM; (**B**) Predictions made by quantitative structure–activity relationship (QSAR) model. Pixels in red correspond to compounds predicted as active; (**C**) Difference of experimental activity matrix and prediction activity matrix. Pixels in red correspond to false positive predictions while those in blue correspond to false negatives. White-colored pixels correspond to correct predictions (20,813 out of 26,580) made by the QSAR model.

Figure 5A,B respectively show the heat maps of the experimental and predicted activity for the model developed using activity specified at *K_d_* < 10 µM.

In the current approach, ANN-based QSAR models were trained to predict the activity of small molecules against a panel of 379 kinases. MCC for the model developed using activity specified at *K_d_* < 3 µM was 0.48, compared to 0.37 for the structure-based models developed by Subramanium and colleagues [13]. These authors developed a computational model to predict the activity of 15 kinase inhibitors against 280 kinase molecules by designing binding site signatures that use 3D X-ray structure information of kinase-inhibitor complexes. Davis and colleagues screened all these inhibitors against a panel of kinases, except one, roscovitine. Table 2 compares the performance of models developed by Subramanium and colleagues for 14 investigated kinase inhibitors to the models developed in this study. The table shows the overall TP, FP, TN and FN kinase-inhibitor pairs.

The performance of the kinase activity models developed by Subramanium and colleagues and those developed here are compared in Table 2. The models developed with activities specified at different cutoffs are reported in the table. Reported are the accuracy, sensitivity and specificity of the models developed for the 15 kinase inhibitors studied by Subramanium and colleagues. The models developed in this study were used for predicting the activity of these 15 kinase inhibitors, and the results are tabulated.

Two models were reported by Subramanium and colleagues for 15 kinase inhibitors at activity cutoffs specified at *K_d_* values of 0.1 and 3 µM. The method involves computing the binding site signature for each inhibitor using a co-crystal structure of the kinase-inhibitor complex. On the basis of the similarity of the binding site signature, the method predicts which other kinases the small-molecule inhibitor can bind to. The models developed in this study predict the activity of small molecules against kinases. Here, the ANN predicts the activity of each inhibitor against 379 kinases on the basis of the chemical structure of the inhibitor. For each kinase, a different threshold of predicted activity is chosen for specifying the activity of small molecules. The models generated in this study using 0.1 µM cutoff values performed worse compared to those reported by Subramanium and colleagues. This is possibly because of the sparsity of data, as there were very few kinase-inhibitor pairs with *K_d_* values of less than 0.1 µM. However, our model generated at cutoff values of 3 µM performed better than those reported by Subramanium and colleagues in terms of high values for MCC, accuracy, and sensitivity. The models reported by these authors have a higher specificity but a very low sensitivity compared to the models reported in this study. Supplementary Table S1 shows the activity prediction for 15 molecules using the models developed here and those developed by Subramanium and colleagues at 3 µM. Our model performs better for highly cross-reactive inhibitors such as staurosporine and VX-680. In general, because the QSAR models have only been trained on type 1 and type 2 kinase inhibitors, their utility is limited to molecules that show inhibitory activity against at least one kinase molecule and that target the ATP binding site.

## 4. Materials and Methods

### 4.1. Kinase Inhibition Profile Dataset

The kinase inhibition dataset published by Davis and colleagues is available in the CHEMBL database. The dataset is available in the CHEMBL document CHEMBL1908390. The scripts for parsing and cleaning the data were written in the R programming language and are available in supplementary information. The protocol capture in the supplementary documents details all the steps necessary for preparing the data for building ANN models.

### 4.2. Software for QSAR Modeling

The software for training QSAR models was developed in-house at the Meiler Laboratory, Department of Chemistry, Vanderbilt University, Nashville, TN, USA. The software is open-source with restrictions (see http://meilerlab.org/bclcommons/license) and is free for academic use; a license is required for commercial use. The executable can be downloaded at http://www.meilerlab.org.

## 5. Conclusions

In this study, QSAR models were developed for predicting the activity of kinase inhibitors against a panel of 379 kinase enzymes. Kinase activity data was reported by Davis and collaborators for 70 inhibitors in terms of *K_d_* values obtained using ATP site-dependent competition binding assays. Five models were developed using activities specified at different *K_d_* cutoff values. Statistical tests suggest that the model using 10 µM as the cutoff for activity has a better predictive ability compared to other models. This model allows for the prediction of the kinase specificity profile for weak binders. A prerequisite to using this model is that a given small molecule to be tested should be active against at least 1 of the 379 tyrosine kinases present in the dataset, as the ANN has been trained only on a small chemical space of known inhibitors. The predictive ability of the model varies significantly; AUC values for 75% of kinases range from 0.5 to 1.0. This model is a good starting point for predicting the selectivity profile of new molecular entities against different kinases. This is especially useful after a computational high-throughput screening of a virtual compound library when compounds need to be prioritized for experimental testing. Ideally, a diverse set of drug-like molecules would be ordered and tested. The selectivity QSAR model developed here could be used for shortlisting compounds by scanning for molecules that are predicted to be selective.

## Figures and Tables

**Table 1:** Quality metrics for five artificial neural network (ANN) models.

Activity Cutoff (µM)	MCC	ACC	SEN	SEL	TP	FP	TN	FN	PPV	NPV
0.1	0.14	68.10	58.99	68.71	971	7787	17097	675	0.11	0.96
0.5	0.26	78.18	54.02	81.26	1620	4410	19121	1379	0.27	0.93
1	0.31	78.99	53.13	83.41	2055	3760	18902	1813	0.35	0.91
3	0.37	78.59	54.20	84.79	2915	3218	17934	2463	0.47	0.88
10	0.42	78.45	57.59	85.27	3765	2944	17048	2773	0.56	0.86

**Table 2:** Comparison of model accuracy.

	Cutoff (µM)	MCC	ACC	SEN	SEL	TP	FP	TN	FN	PPV	NPV
Subramanium and Colleagues [22]	0.1	0.35	87.3	52.3	90.5	185	370	3521	169	0.33	0.95
	3	0.37	81.0	36.6	93.6	342	213	3098	592	0.61	0.83
BCL::Cheminfo	0.1	0.24	67.7	75.4	67.0	340	1727	3507	111	0.16	0.97
	3	0.48	78.6	74.5	79.8	944	891	3526	324	0.51	0.91
	10	0.52	79.3	73.8	81.4	1174	762	3332	417	0.61	0.88

## Supplementary Materials

The following are available online.
