# Supplementary material for: Quantitative Structure–Activity Relationship Modeling of Kinase Selectivity Profiles
Source: Molecules. 2017 Sep 19;22(9):1576. doi: 10.3390/molecules22091576 (PMC6151389; doi:10.3390/molecules22091576)
Supplement: Supplementary file 1 [file molecules-22-01576-s001.pdf]

molecules

MDPI

Table 1 Molecule wise comparison of predictive ability of models developed by Subramanium et al. and developed in this study. The active molecules are indicated at cutoff value of $K_D = 3$ μM.

Inhibitor Type	Molecule	Subramanium et al (3 μM Models)							3 μM QSAR Models						
		ACC	SEN	SEL	TP	FP	TN	FN	ACC	SEN	SEL	TP	FP	TN	FN
Type I	Dasatinib	81	44	96	36	9	193	45	78	47	89	48	30	247	54
	Erlotinib	85	19	97	8	8	233	34	93	0	93	0	25	354	0
	Gefitinib	80	52	82	11	47	215	10	79	66	81	29	65	270	15
	LY-333531	77	43	83	18	42	199	24	77	63	80	46	60	246	27
	Roscovitine	96	0	99	0	2	271	10	–	–	–	–	–	–	–
	SB-203580	89	10	99	3	3	249	28	88	18	94	6	19	326	28
	Staurosporine	46	39	97	96	1	33	153	83	87	54	289	21	25	44
	VX-680	64	3	100	3	0	178	102	68	76	64	100	88	159	32
	VX-745	97	20	100	2	0	273	8	73	89	73	8	100	270	1
Type II	BIRB-796	83	10	98	5	5	230	43	67	84	64	42	118	211	8
	Flavopiridol	78	2	97	1	7	221	54	71	78	69	75	89	194	21
	Imatinib	94	68	96	13	11	253	6	86	68	87	15	46	311	7
	Lapatinib	100	100	100	3	0	280	0	84	67	84	4	60	313	2
	Sorafenib	84	35	96	19	9	219	36	78	81	77	55	72	239	13
	Sunitinib	63	76	45	124	66	54	39	78	80	75	181	38	115	45
